# Subclinical Hypothyroidism in Advanced Chronic Kidney Disease Patients: Prevalence and Associated Factors

**DOI:** 10.1155/2022/1077553

**Published:** 2022-05-17

**Authors:** Javier Reque Santivañez, Beatriz Garcia Peris, Nayara Panizo Gonzalez, Alejandro Perez Alba, Luis D'Marco, Eládio Collado Boira

**Affiliations:** ^1^Hospital General Universitario de Castellón, Nephrology Department, Valencia, Spain; ^2^Hospital Clínico Universitario de Valencia, INCLIVA, Nephrology Department, Valencia, Spain; ^3^Departamento de Medicina y Cirugía, Facultad Ciencias de la Salud, Universidad Cardenal Herrera-CEU, Alfara del Patriarca, Spain; ^4^Universidad Jaime I Castellón, Valencia, Spain

## Abstract

**Introduction:**

Renal function and thyroid metabolism are tightly related. However, evidence about subclinical hypothyroidism prevalence in patients with chronic kidney disease and its related factors is scarce.

**Objectives:**

Our aim is to analyze subclinical hypothyroidism prevalence and its related factors in patients with advanced chronic kidney disease. *Materials and methods*. Nondialysis-dependent patients with chronic kidney disease at stages 3 to 5 were included. Other inclusion criteria were age above 18 years and clinical stability. Patients with diagnosed thyroid illnesses were excluded. Subclinical hypothyroidism was defined as thyroid stimulating hormone (TSH) > 5.3 mU/L, with free thyroxine 4 (FT4) between 0.54 and 1.24 ng/dl. Filiation data, comorbidities, and routine blood and urine test results were registered.

**Results:**

A total of 299 patients were included. Of them, 184 (61.5%) were men. The mean age was 71 ± 13 years old. The mean glomerular filtration rate (CKD-EPI) was 22 ± 9 ml/min/1.73 m^2^. According to chronic kidney disease stages, global distribution of patients was as follows: Stage 3, 67 patients (22.4%); Stage 4, 155 patients (51.8%); and Stage 5, 77 patients (25.8%). We found subclinical hypothyroidism in 54 (18.1%) patients. According to chronic kidney disease stages, distribution of affected patients was as follows: Stage 3, 9 patients (13%); Stage 4, 25 patients (16.1%); and Stage 5, 20 patients (26%). Differences among stages were statistically significant. By univariate analysis, factors related with subclinical hypothyroidism were as follows: age RR 1.048 (95% CI 1.019–1.078; *p*=0.001), hypertension RR 2.705 (95% CI 1.026–7.130; *p*=0.04), glomerular filtration rate RR 0.962 (95% CI 0.929–0.996; *p*=0.03), and proteinuria higher than 1 gram/day RR 2.387 (95% CI 1.303–4.374; *p*=0.005). By multivariate analysis adjusted by age, hypertension, glomerular filtration rate, proteinuria, diabetes, and cardiovascular disease history, only age RR 1.016 (95% CI 1.009–1.028; *p*=0.04) and glomerular filtration rate RR 0.963 (95% CI 0.930–0.997; *p*=0.03) preserved their independent association with subclinical hypothyroidism.

**Conclusions:**

Subclinical hypothyroidism prevalence in patients with chronic kidney disease is high and increases with renal disease severity. Factors independently related to subclinical hypothyroidism are age and glomerular filtration rate.

## 1. Introduction

Chronic kidney disease (CKD) has risen dramatically in recent years becoming a truepublic health problem worldwide [1]. A recent population-based study estimates the prevalence of CKD in Spain at around 15.1% [2]. The poor prognosis in terms of morbidity and mortality of CKD patients has been extensively documented [1, 3]. Thus, at least in part, poor prognosis observed in these patients could be associated with the influence of decreased kidney function on the rest of the body's homeostatic systems.

In this regard, despite the fact that some patients with subclinical hypothyroidism (SH) may have clinical manifestations derived from this mild thyroid hypofunction, SH is a primarily biochemical diagnosis based on the detection of high concentrations of thyroid-stimulating hormone (TSH) in conjunction with a low or normal concentration of free thyroxine (fT4) [4]. Of note, there is evidence that SH is associated with higher cardiovascular mortality in the general population [5, 6]. Furthermore, there is evidence suggesting that the prevalence of SH in patients with CKD is high [7]; however, the evidence and the percentage of advanced CKD-affected patients are quite low (0.3%) and remain scarce. The objective of this study is to analyze the prevalence of SH in patients with advanced CKD as well as the factors associated with this condition.

## 2. Methods

This cross-sectional study includes patients with advanced CKD evaluated in our outpatient clinic at Hospital General Universitario in Castellón between January and June 2018. The inclusion criteria were as follows: age above 18 years and absence of hospital admissions or emergency care in the last 3 months. Patients diagnosed with hypothyroidism and on hormone replacement therapy were excluded.

We collected filiation data and comorbidity history from the electronic medical record. The cardiovascular history collected was as follows: cardiac diseases (heart failure, angina, and acute myocardial infarction), strokes, and peripheral vascular disease episodes. Likewise, routine analytical parameters and thyroid function were collected.

The presence of CKD was defined according to the current Kidney Disease: Improving Global Outcomes (KDIGO) guidelines [8], using the estimated glomerular filtering rate (eGFR) calculated using the CKD-Epidemiology Collaboration (CKD-EPI) equation.

We define subclinical hypothyroidism as a TSH value > 5.3 mU/L with fT4 values between 0.54 and 1.24 ng/dl measured by chemiluminescence with a UniCel DxI 800 Immunoassay System autoanalyzer (Beckman Coulter, Inc.).

### 2.1. Statistical Analysis

The data were analyzed using the SPSSv20.0 program (IBM, Munich, Germany); the variables of normal distribution are expressed as mean and standard deviation and the nonparametric variables as median and interquartile range. We determined the differences between groups using the Student's *t*-test, chi-square test, and Mann–Whitney U test appropriately. To analyze the variables related to subclinical hypothyroidism, we performed a logistic regression, including those that were initially significant in the univariate analysis in the multivariate model. Statistical significance was established at a value of *p* < 0.05.

## 3. Results

A total of 299 patients were included in the study, of these 184 (61.5%) were male, with a mean age of 71 ± 13 years. The mean eGFR was 22 ± 9 ml/min/1.73 m^2^. The distribution of patients according to CKD stages was as follows: Stage 3, 67 patients (22.4%); Stage 4, 155 patients (51.8%); and Stage 5, 77 patients (25.8%). The rest of the baseline characteristics are shown in Table 1.

**Figure 1:**
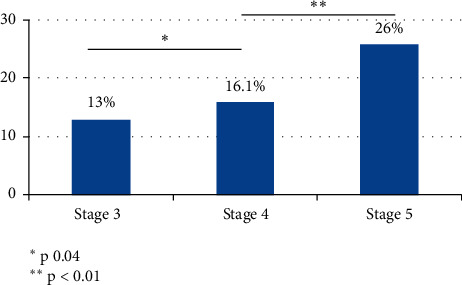
Prevalence of subclinical hypothyroidism according to the CKD stage.

We found a biochemical pattern compatible with SH in 54 (18.1%) patients. The prevalence of SH according to CKD stages was as follows: Stage 3, 9 patients (13%); Stage 4, 25 patients (16.1%); and Stage 5, 20 patients (26%), the difference between stages being statistically significant, as shown in Figure 1. When we separate the patients according to the presence or absence of SH, we found that the former had a significantly older age, more prevalence of high blood pressure, lower GFR, and higher proteinuria.

In a univariate analysis, the factors associated with subclinical hypothyroidism were as follows: age RR 1.048 (95% CI 1.019–1.078; *p*=0.001), the presence of hypertension RR 2.705 (95% CI 1.026–7.130; *p*=0.04), GFR RR 0.962 (95% CI 0.929–0.996; *p*=0.03), and proteinuria levels greater than 1 gr/day RR 2.377 (95% CI 1.303–4.374; *p*=0.005).

In a multivariate analysis adjusted for age, hypertension, GFR, proteinuria, diabetes, and cardiovascular history, they only maintained their independent association with subclinical hypothyroidism for age RR 1.016 (95% CI 1.009–1.028; *p*=0.04) and the GFR RR 0.963 (95% CI 0.930–0.997; *p*=0.03) (Table 2).

## 4. Discussion

The prevalence of SH in our study is slightly higher (18.1%) than that reported for the general population, in which the ranges vary between 3% and 15%. Of note, for the general population, the main predisposing factors are age, male gender, and iodine deficiency [9, 10]. Of interest, our findings show an increase in the prevalence of SH as the severity of CKD increases. Similar findings were reported by Chonchol et al. in a large sample size (3,089 patients) investigation [7]. In this study, the prevalence of SH was 17.9% in patients with eGFR <60 ml/min/1.73 m^2^. However, it should be noted that only 11 patients presented eGFR <30 ml/min/1.73 m^2^ (0.7% of the sample). Similarly, in a study that included 14,623 patients, the prevalence of hypothyroidism (both clinical and subclinical) in patients with GFR <30 ml/min/1.73 m^2^ was 23.1% [11].

Different hypotheses have been postulated to explain the increased prevalence of SH in CKD patients such as an alteration in iodine metabolism, decreased peripheral sensitivity to the thyroid hormone, or a higher prevalence of autoimmune thyroiditis [12]. Nonetheless, the exact mechanisms responsible for this finding are not entirely understood, and it is likely to have a multifactorial etiology. In fact, it has been proved that iatrogenic hypothyroidism significantly increases serum creatinine levels and reversibly impairs eGFR, while treatment with rhTSH enhances renal function in euthyroid patients, supporting the existence of an influence of the TSH level on renal function [13].

In the general population, age has been found as an independent predictor of SH [14, 15]. In studies from the institutionalized elderly population, a higher prevalence of SH (5.2% vs. 1.1%) was found in patients older than 80 years compared to younger patients [16].

We found a higher percentage of patients with proteinuria (>1 gr/day) associated with SH. There is evidence that the presence of proteinuria could contribute to the urinary loss of T4L, maintaining the patients without symptoms at the expense of the increased production of TSH [17]. However, in the adjusted analysis, proteinuria loses statistical significance. The number of patients included and the fact that the cut-off point was established at 1 gram of proteinuria per day could explain this finding.

The risk of conversion from SH to clinical hypothyroidism is relatively low, approximately 2 to 6% per year [18, 19]. However, in a meta-analysis of 11 prospective studies, including more than 55,000 patients, a significantly higher risk of coronary heart disease could be demonstrated in SH-affected patients [6]. Other studies associate SH with congestive heart failure [20] and stroke [6]. Some data suggest that thyroid dysfunction might be implicated in the pathogenic pathway which links micro-inflammation to survival in peritoneal dialysis patients [21]. Regarding the impact on the long-term prognosis and the high prevalence of SH in CKD patients, the routine determination of thyroid hormone concentrations in these populations should be justified.

We must recognize some limitations in our study, such as sample size. Although there are studies with a larger population, this is the only one that includes a significant number of patients with advanced CKD, so we believe that it is representative of this population. No data have been collected regarding the medication used by the patients; it is possible that some drugs may influence thyroid function, and finally, anti-TPO titers have not been determined, which could certainly clarify the obtained results.

## 5. Conclusion

The prevalence of SH in CKD patients is high. This prevalence increases as CKD worsens. Age and eGFR are independent predictors of SH. More studies are needed to determine the impact of SH on the long-term prognosis of patients and to be able to assess the benefits of hormone replacement therapy in the CKD population.

## Figures and Tables

**Table 1:** Clinical characteristics of the study population.

	All, 299 (100%)	SH present, 54 (18.1%)	SH absent, 245 (81.9%)	*p*
Age (years)	71 ± 13	76 ± 11	69 ± 13	<0.001
Gender (male)	184 (61.5%)	33 (61.1%)	151 (61.6%)	0.9
Hypertension	241 (80.6%)	49 (90.7%)	192 (78.4%)	0.03
Diabetes mellitus	117 (39.1%)	29 (38.9%)	96 (39.2%)	0.9
Dyslipidemia	219 (73.2%)	42 (77.8%)	177 (77.2%)	0.06
CV disease history	137 (45.8%)	29 (53.7%)	108 (44.1%)	0.05
Hemoglobin (g/dL)	12.2 ± 1.4	12.3 ± 1.2	12.1 ± 1.4	0.3
Parathyroid hormone (pg/mL)	174 ± 96	185 ± 115	172 ± 91	0.08
TSH (mU/L)	3.1 ± 1.6	6 ± 0.8	2.5 ± 1	<0.001
fT4 (ng/dL)	1 ± 0.6	0.9 ± 0.1	0.9 ± 0.6	0.8
CKD EPI (ml/min/1.73 m^2^)	22 ± 9	19 ± 9	22 ± 9	0.02
Proteinuria > 1 gram per day	90 (30.1%)	25 (46.3%)	65 (26.5%)	0.004

Patients were divided according to the presence of subclinical hypothyroidism. CV: cardiovascular; TSH: thyroid-stimulating hormone; fT4: free thyroxine.

**Table 2:** Logistic regression analyses for variables associated with subclinical hypothyroidism.

	Unadjusted		Adjusted^*∗*^	
	RR (95% CI)	*p*	RR (95% CI)	*p*
Age (1 year)	1.048 (1.019–1.078)	0.001	1.016 (1.009–1.028)	0.04
Hypertension	2.705 (1.026–7.130)	0.04		
eGFR (1 mL/min 1.73 m^2^)	0.962 (0.929–0.996)	0.03	0.963 (0.930–0.997)	0.03
Proteinuria > 1 g/day	2.387 (1.303–4.374)	0.005		

^*∗*^Multivariate model includes all variables that were associated with subclinical hypothyroidism by univariate analyses. eGFR: estimated glomerular filtration rate.

## Data Availability

Data are available upon request.
